# Association of Formal and Informal Advance Care Planning with Multidimensional Health Literacy in Patients Undergoing Hemodialysis

**DOI:** 10.34067/KID.0000001243

**Published:** 2026-05-26

**Authors:** Tomo Suzuki, Tatsunori Toida, Yu Munakata, Yusuke Kanakubo, Mamiko Ukai, Ryohei Inanaga, Takumi Toishi, Tetsuro Aita, Atsuro Kawaji, Masatoshi Matsunami, Tadao Okada, Noriaki Kurita

**Affiliations:** 1 Department of Clinical Epidemiology, Graduate School of Medicine, Fukushima Medical University, Fukushima, Japan; 2 Department of Nephrology, Kameda Medical Center, Chiba, Japan; 3 Division of Nephrology, Toho University Ohashi Medical Center, Tokyo, Japan; 4 Munakata Clinic, Chiba, Japan; 5 Chikuseikai Munakata Clinic, Tokyo, Japan; 6 Tessyoukai Kameda Family Clinic Tateyama, Chiba, Japan; 7 Division of Clinical Epidemiology, Research Center for Medical Sciences, The Jikei University School of Medicine, Tokyo, Japan; 8 Department of Nephrology, Shin-Yurigaoka General Hospital, Kanagawa, Japan; 9 Department of General Internal Medicine and Family Medicine, Fukushima Medical University, Fukushima, Japan; 10 Department of Innovative Research and Education for Clinicians and Trainees (DiRECT), Fukushima Medical University Hospital, Fukushima, Japan; 11 Department of Preventive Medicine, Tokai University School of Medicine, Kanagawa, Japan

**Keywords:** dialysis, hemodialysis, outcomes

## Abstract

**Key Points:**

- Higher critical health literacy was associated with informal advance care planning, while higher total health literacy was associated with both informal and formal advance care planning in patients receiving hemodialysis.
- Patients unsure about treatment preferences had lower total health literacy, and lack of opportunity was the most common barrier to advance care planning engagement.
- Enhancing health literacy and providing structured opportunities for advance care planning may support patient-centered decision making in dialysis care.

**Background:**

Although qualitative studies suggest that health literacy (HL) may influence engagement in advance care planning (ACP) among patients undergoing dialysis, this association has not been quantitatively examined. We investigated whether multidimensional HL is associated with participation in informal and formal ACP and end-of-life care preferences.

**Methods:**

This multicenter cross-sectional study included adult patients receiving hemodialysis from six facilities in Japan. Participation in informal and formal ACP was assessed using a questionnaire, and HL was measured with the Functional, Communicative, and Critical Health Literacy scale. Associations between HL and ACP participation were analyzed using logistic regression models. We also examined patients' end-of-life care preferences in the event of serious future health deterioration.

**Results:**

Among 651 eligible patients, 466 were included in the analysis (median age, 71 years; 35% women). Informal ACP participation was reported by 52% of patients, whereas formal ACP participation was 5%. Higher critical HL was independently associated with informal ACP participation (odds ratio [OR], 1.93; 95% confidence interval [CI], 1.25 to 3.00), while higher total HL was associated with formal ACP participation (OR, 4.18; 95% CI, 1.63 to 10.77). Women and diabetes were associated with informal ACP and female sex with formal ACP. Patients who reported being unsure about future treatment preferences had lower total HL and were younger (total HL: OR, 0.63; 95% CI, 0.42 to 0.95; age: OR, 0.79; 95% CI, 0.66 to 0.95). The most common reason for not engaging in ACP discussions was a lack of opportunity to discuss ACP (71/151).

**Conclusions:**

Critical HL was associated with informal ACP participation, whereas total HL was associated with formal ACP among patients undergoing hemodialysis. In addition, the most frequently reported barrier to ACP engagement was a lack of opportunity for discussion, highlighting the need to both enhance HL and establish structured opportunities for ACP to support patient-centered decision making.

## Visual Abstract

**Figure:**
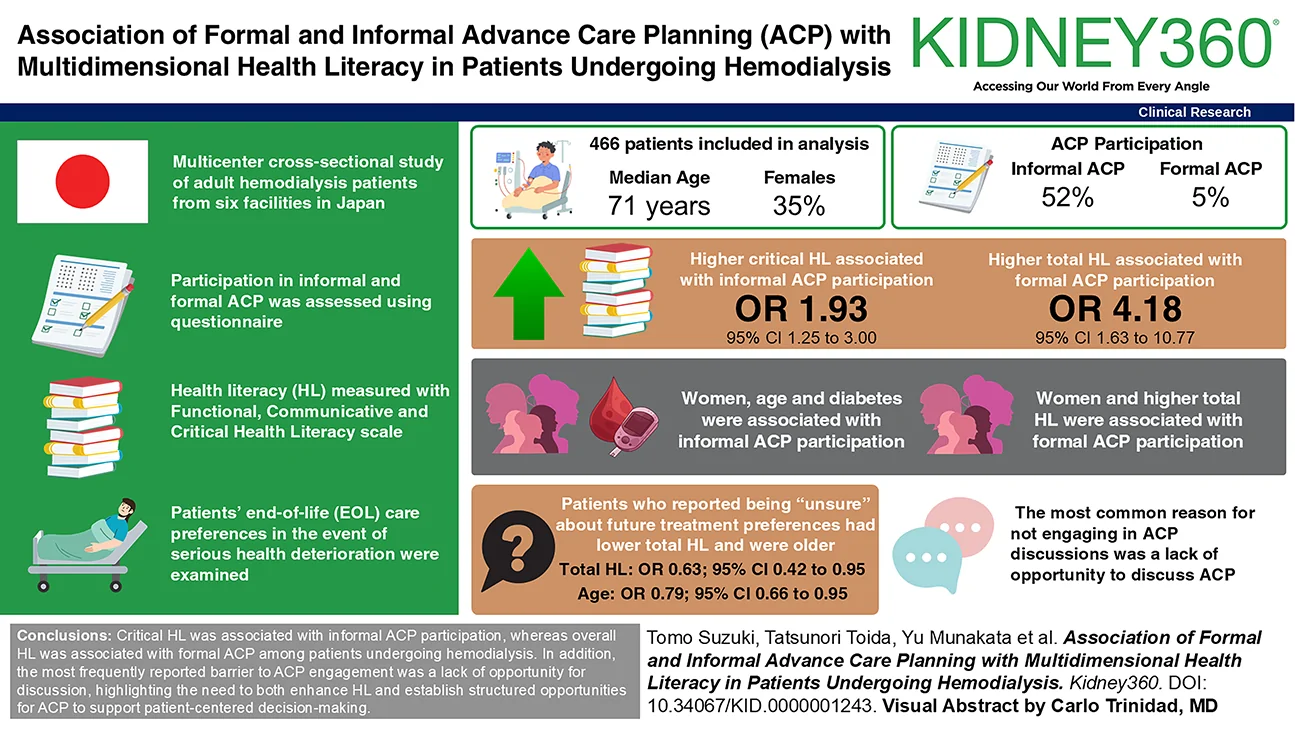

## Introduction

Advance care planning (ACP) is a process that prepares for potential loss of decision-making capacity by clarifying and communicating patients' values, life goals, and preferences for future care.^1^ It is an integral component of person-centered care for patients receiving dialysis, who face a higher mortality risk than the general population.^2^ ACP may be formal, involving structured discussions and the completion of advance directives (ADs),^3,4^ or informal, consisting of conversations with family or health care professionals about end-of-life (EOL) values and preferences that are not formally documented. In Japan, where ADs are neither legally binding nor widely implemented, ACP is primarily promoted through ongoing discussions with family members and health care professionals about future care.^5^ Randomized trials among patients receiving hemodialysis have shown that health care professional–facilitated ACP dialogs may improve preparedness for EOL decision making.^6^ However, the clinical implications of inadequate ACP are substantial. In settings such as home care and long-term care facilities, insufficient ACP has been associated with poorer quality of dying and reduced psychological preparedness among family caregivers.^7,8^ Nevertheless, ACP remains difficult to implement in routine practice among patients receiving dialysis; in a Japanese survey, fewer than 11% of elderly patients on dialysis reported engaging in informal ACP with health care professionals.^9^ Identifying modifiable barriers to ACP is therefore essential.

Health literacy (HL)—the ability to access, process, and use health information—has been proposed as a potential enabler for ACP in qualitative research but has not been adequately examined quantitatively. HL is a multidimensional construct commonly conceptualized as comprising functional, communicative, and critical HL domains.^10,11^ Functional HL refers to basic reading and numeracy skills required to process health information, communicative HL involves the ability to obtain information through interaction and apply it to changing situations, and critical HL refers to the ability to critically appraise information and make informed decisions. These domains are often conceptualized as hierarchical and complementary, with functional HL forming the foundation upon which communicative and critical HL is build, and higher order domains contributing more directly to understanding and decision making. This framework enables a comprehensive assessment of HL relevant to health decision making. Each domain has been suggested to play an important role in sustaining care among patients receiving dialysis. One qualitative study proposed that limited functional HL may hinder familiarity with ACP-related terms (*e.g*., prognosis), restricted communicative HL may reduce opportunities for discussion with family and health care professionals, and inadequate critical HL may impede EOL decision making.^12^ However, whether multidimensional HL is associated with ACP engagement in patients undergoing dialysis remains unclear.

Therefore, the aim of this study was to examine the associations between multidimensional HL and participation in both formal and informal ACP, as well as to EOL care preferences, in a multicenter cross-sectional study.

## Methods

### Study Design and Population

This multicenter cross-sectional study was conducted at six medical facilities providing outpatient hemodialysis services. The study was reported in accordance with the Strengthening the Reporting of Observational Studies in Epidemiology guidelines. This study followed the Declaration of Helsinki and was approved by the Ethical Review Board of Fukushima Medical University (number: ippan 2021-292). Inclusion criteria were as follows: (*1*) adult patients with kidney failure receiving hemodialysis, including those on combination therapy with peritoneal dialysis (*i.e*., concurrent use of peritoneal dialysis with intermittent hemodialysis to supplement fluid or solute removal),^13^ (*2*) receiving scheduled dialysis treatment as prescribed at a participating institution, and (*3*) ability to understand and complete the questionnaire. Written consent was obtained from all participants. Data were collected between April 2022 and February 2023. Patients received a payment of 500 yen (approximately USD $4), which represents a modest, noncoercive token of appreciation and is consistent with standard practice in questionnaire-based studies in Japan.

### Measurements

The full questionnaire for ACP-related measures is provided in Supplemental Item 1. The questionnaire was reviewed by the research team to ensure clarity before administration. All questionnaires were administered in Japanese.

### Outcomes: ACP Participation and Treatment Preference

ACP participation and treatment preferences were assessed using items adapted from previous US studies in patients with kidney failure.^14^ These items use plain language and are designed as single-question measures rather than multi-item scales. The items for ACP participation and treatment preferences were translated into Japanese by the research team to ensure conceptual clarity; however, formal forward–backward translation procedures were not conducted.

ACP participation was assessed on the basis of whether participants had considered and/or discussed preferences for future medical care in the event of serious illness and inability to communicate. Responses were categorized as:

- *No ACP*: no consideration or consideration without discussion.
- *Informal ACP*: discussion with family or health care professionals.
- *Formal ACP*: completion of ADs.

Treatment preference was assessed on the basis of which preferred approach participants indicate to future medical care in the context of serious illness. Responses were categorized as:

- *Longevity-focused care*: life-prolonging treatment.
- *Comfort-focused care*: symptom-oriented treatment.
- *Unsure*.

#### Reasons for Not Discussing ACP

Participants who had considered ACP but had not discussed it were asked to select reasons from predefined options (multiple responses allowed), including lack of opportunity, lack of knowledge, absence of a discussion partner, and personal reluctance. All ACP-related measures were assessed using single-question items with predefined response options. Given their descriptive and nonscale-based nature, formal psychometric evaluation was not applicable.

### Exposure: Multidimensional HL

HL was assessed using the Functional, Communicative, and Critical Health Literacy scale (Supplemental Table 1).^10^ This instrument was originally developed and validated in Japanese populations; therefore, no cultural adaptation or translation was required for this study. This 14-item instrument includes three domains: functional, communicative, and critical HL. Each item is rated on a four-point Likert scale, ranging from one (never) to four (often). The functional HL items were reverse-coded. Domain scores and total HL were calculated as mean values (range, 1–4), with higher scores indicating higher HL. The Functional, Communicative, and Critical Health Literacy demonstrated good validity and internal consistency in Japanese patients with type 2 diabetes (Cronbach *α*: 0.84, 0.77, and 0.65 for functional, communicative, and critical HL, respectively)^10^ and has been widely used in studies involving patients with chronic diseases, including those undergoing dialysis.^15^

### Measurement of Covariates

Confounding variables were selected on the basis the literature and expert medical knowledge and included those suspected to affect HL and participation in formal and informal ACP. The variables included age, sex, education level, household income, medical history with diabetes, and malignancy. The definition of each covariate is provided in Supplemental Item 2. The questionnaire was administered at each facility. Participants were instructed to complete the questionnaire at home by themselves. However, assistance (*e.g*., reading support) was not formally assessed. Completed questionnaires were returned to the dialysis facility at the next visit.

### Statistical Analyses

All statistical analyses were performed using Stata/SE, version 18.0 (StataCorp, College Station, TX). Patient characteristics are described as medians and interquartile ranges for continuous variables and as frequencies and percentages for categorical variables. Associations between HL and ACP participation were examined using logistic regression models, separately for informal and formal ACP. In each analysis, two models were constructed: one including HL subdomains (functional, communicative, and critical HL) and another including total HL. To examine the association between HL and reporting unsure preferences for future medical care, additional logistic regression analyses were conducted using the same modeling approach. Unadjusted and multivariable-adjusted odds ratios (AORs) were estimated for total HL and each HL subdomain. All covariates described earlier were entered simultaneously into the multivariable-adjusted models. Given that these covariates represent conceptually distinct constructs, variables were retained in the models regardless of potential correlations to ensure appropriate confounder adjustment. Missing data were handled using multiple imputation with chained equations (20 datasets), assuming data were missing at random. Formal analyses were conducted by the first author and independently verified by an experienced epidemiologist (N. Kurita). For all analyses, a two-tailed *P* value of <0.05 was considered statistically significant.

## Results

### Study Flow and Participant Characteristics

**Figure 1:**
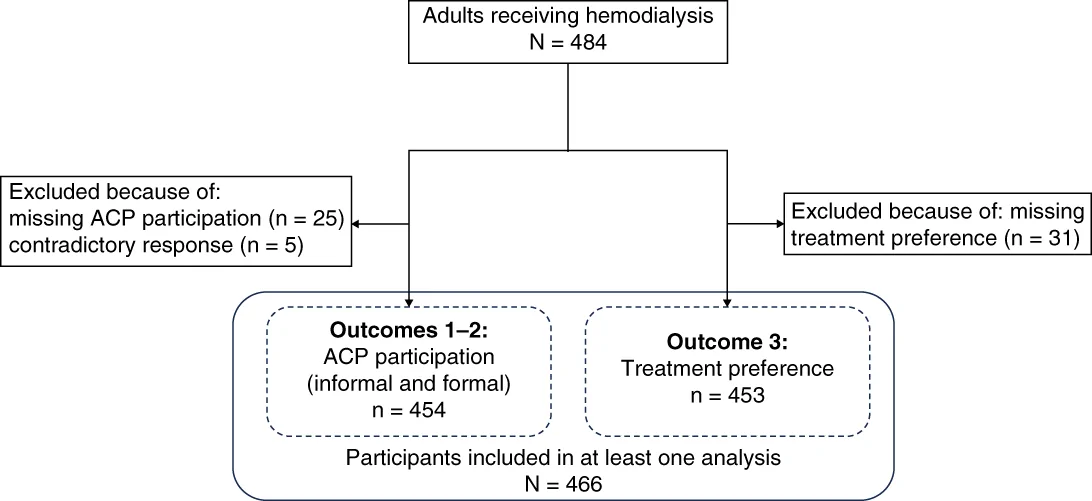
**Participant flow and analytic sample selection.** ACP, advance care planning.

Of the 651 patients undergoing hemodialysis at six centers, 484 (74%) participated in the study. Among them, 30 were excluded because of missing or invalid data on ACP participation (*n*=25 missing and *n*=5 invalid responses, such as logically contradictory answers), and 31 had missing data on treatment preferences. After excluding 18 patients whose data on both ACP participation and treatment preferences were not analyzable, 466 patients were included in the final analysis (Figure 1). Participant characteristics are summarized in Table 1. The median age was 71 years (interquartile range, 63–78), and 35% were women. Diabetic nephropathy was the most common cause of kidney failure (38%). Approximately half reported an annual household income <3,000,000 yen, and 13% had attained a university or graduate-level education. Regarding ACP participation, 52% had engaged in informal ACP, while 5% had participated in formal ACP.

**Table 1:** Participant characteristics (*N*=466)

Characteristic	Overall (*N*=466)	Missing, *n*
**Demographic**		
Age, yr	71 (63–78)	28
Women	159 (35)	13
**Cause of kidney failure**		0
Diabetic nephropathy	175 (38)	
Chronic GN	107 (23)	
Nephrosclerosis	62 (13)	
Polycystic kidney disease	13 (3)	
Others	72 (15)	
Unknown	37 (8)	
**Dialysis modality**		0
Hemodialysis	450 (97)	
Combined hemodialysis and peritoneal dialysis	16 (3)	
**Comorbidities**		
Diabetes	200 (43)	3
Malignancy	59 (13)	1
**Household income**		36
<3,000,000 yen (low)	232 (54)	
3,000,000 to <5,000,000 yen (medium)	112 (26)	
≥5,000,000 yen (high)	86 (20)	
**Education**		20
Junior high school or less	108 (24)	
High school	217 (49)	
University/graduate school	59 (13)	
Others	62 (14)	
**ACP-related characteristics**		
ACP participation		
*Informal ACP*	235 (52)	12
*Formal ACP*	24 (5)	12
**Treatment preferences under serious illness**		13
Longevity-focused	18 (4)	
Comfort-focused	299 (66)	
Unsure	136 (30)	

Data are presented as median (interquartile range) for continuous variables and *n* (%) for categorical variables. Missing values are reported for each variable. Informal advance care planning was defined as discussion with family or physicians, regardless of documentation, and formal advance care planning as completion of advance directives. ACP, advance care planning.

### Association between Formal and Informal ACP Participation and HL

Table 2 presents the association between multidimensional HL and informal ACP participation (left panel). Higher critical HL was significantly associated with informal ACP participation (AOR, 1.93; 95% confidence interval [CI], 1.25 to 3.00; *P* = 0.003), whereas functional and communicative HL were not (AOR, 0.81; 95% CI, 0.61 to 1.07; *P* = 0.14; and AOR, 1.15; 95% CI, 0.75 to 1.76; *P* = 0.51; respectively). Diabetes mellitus, age, and women were associated with informal ACP participation (diabetes: AOR, 1.53; 95% CI, 1.02 to 2.29; *P* = 0.04; age: AOR, 1.24; 95% CI, 1.04 to 1.48; *P* = 0.02; women: AOR, 1.56; 95% CI, 1.01 to 2.41; *P* = 0.04). Total HL was also significantly associated with informal ACP participation (per one-point increase: AOR, 1.80; 95% CI, 1.23 to 2.63; *P* = 0.003; Table 2, right panel).

**Table 2:** Associations between health literacy and informal advance care planning participation (*n*=454)

Variables	Multidimensional HL Model		Total HL Model	
	Unadjusted OR (95% CI), *P* Value	AOR (95% CI), *P* Value	Unadjusted OR (95% CI), *P* Value	AOR (95% CI), *P* Value
Total HL (per one-point increase)	—	—	1.55 (1.10 to 2.17), *P* = 0.01	1.80 (1.23 to 2.63), *P* = 0.003
Functional HL (per one-point increase)	0.78 (0.60 to 1.01), *P* = 0.06	0.81 (0.61 to 1.07), *P* = 0.14	—	—
Communicative HL (per one-point increase)	1.11 (0.73 to 1.68), *P* = 0.64	1.15 (0.75 to 1.76), *P* = 0.51	—	—
Critical HL (per one-point increase)	1.84 (1.20 to 2.81), *P* = 0.005	1.93 (1.25 to 3.00), *P* = 0.003	—	—
Age (per 10-yr increase)	—	1.24 (1.04 to 1.48), *P* = 0.02	—	1.19 (1.005 to 1.42), *P* = 0.04
Women (versus men)	—	1.56 (1.01 to 2.41), *P* = 0.04	—	1.60 (1.04 to 2.44), *P* = 0.03
**Comorbidities**				
Diabetes	—	1.53 (1.02 to 2.29), *P* = 0.04	—	1.58 (1.06 to 2.35), *P* = 0.03
Malignancy	—	1.09 (0.60 to 2.00), *P* = 0.77	—	1.08 (0.60 to 1.95), *P* = 0.80
**Household income**				
<3,000,000 yen (low)	—	Reference	—	Reference
3,000,000 to <5,000,000 yen (medium)	—	1.24 (0.77 to 2.00), *P* = 0.38	—	1.22 (0.76 to 1.96), *P* = 0.41
≥5,000,000 yen (high)	—	1.31 (0.77 to 2.24), *P* = 0.32	—	1.27 (0.75 to 2.13), *P* = 0.38
**Education**				
Junior high school or less	—	Reference	—	Reference
High school	—	0.88 (0.52 to 1.48), *P* = 0.62	—	0.74 (0.45 to 1.24), *P* = 0.26
University/graduate school	—	0.92 (0.44 to 1.92), *P* = 0.82	—	0.81 (0.39 to 1.66), *P* = 0.56
Others	—	1.12 (0.56 to 2.23), *P* = 0.76	—	1.00 (0.51 to 1.98), *P* = 1.00

Adjusted odds ratios were estimated using multivariable logistic regression models including all covariates simultaneously. Health literacy scores (range, 1–4) represent mean values; odds ratios indicate the change associated with a one-point increase in these mean scores. Categorical variables are presented relative to the reference group. — indicates not applicable. AOR, adjusted odds ratio; CI, confidence interval; HL, health literacy; OR, odds ratio.

The association between total HL and formal ACP participation is presented in Table 3 (right panel). Higher total HL and women were associated with formal ACP participation (total HL, per one-point increase: AOR, 4.18; 95% CI, 1.63 to 10.77; *P* = 0.003; women: AOR, 2.92; 95% CI, 1.14 to 7.50; *P* = 0.03). By contrast, none of the HL subdomains were significantly associated with formal ACP participation (Table 3, left panel).

**Table 3:** Associations between health literacy and formal advance care planning participation (*n*=454)

Variables	Multidimensional HL Model		Total HL Model	
	Unadjusted OR (95% CI), *P* Value	AOR (95% CI), *P* Value	Unadjusted OR (95% CI), *P* Value	AOR (95% CI), *P* Value
Total HL (per one-point increase)	—	—	3.17 (1.36 to 7.40), *P* = 0.008	4.18 (1.63 to 10.77), *P* = 0.003
Functional HL (per one-point increase)	1.19 (0.65 to 2.17), *P* = 0.57	1.31 (0.69 to 2.48), *P* = 0.41	—	—
Communicative HL (per one-point increase)	1.58 (0.61 to 4.06), *P* = 0.35	1.70 (0.65 to 4.42), *P* = 0.28	—	—
Critical HL (per one-point increase)	1.68 (0.64 to 4.37), *P* = 0.29	1.89 (0.70 to 5.14), *P* = 0.21	—	—
Age (per 10-yr increase)	—	1.43 (0.93 to 2.18), *P* = 0.10	—	1.41 (0.92 to 2.17), *P* = 0.12
Women (versus men)	—	2.88 (1.12 to 7.41), *P* = 0.03	—	2.92 (1.14 to 7.50), *P* = 0.03
**Comorbidities**				
Diabetes	—	1.56 (0.62 to 3.90), *P* = 0.35	—	1.59 (0.64 to 3.98), *P* = 0.32
Malignancy	—	0.25 (0.03 to 1.97), *P* = 0.19	—	0.25 (0.03 to 1.97), *P* = 0.19
**Household income**				
<3,000,000 yen (low)	—	Reference	—	Reference
3,000,000 to <5,000,000 yen (medium)	—	0.61 (0.21 to 1.78), *P* = 0.37	—	0.61 (0.21 to 1.78), *P* = 0.37
≥5,000,000 yen (high)	—	0.29 (0.06 to 1.33), *P* = 0.11	—	0.29 (0.06 to 1.31), *P* = 0.11
**Education**				
Junior high school or less	—	Reference	—	Reference
High school	—	1.42 (0.43 to 4.66), *P* = 0.56	—	1.32 (0.41 to 4.29), *P* = 0.64
University/graduate school	—	1.10 (0.20 to 6.11), *P* = 0.92	—	1.04 (0.19 to 5.77), *P* = 0.96
Others	—	0.81 (0.16 to 3.98), *P* = 0.79	—	0.76 (0.15 to 3.69), *P* = 0.73

Adjusted odds ratios were estimated using multivariable logistic regression models including all covariates simultaneously. Health literacy scores (range, 1–4) represent mean values; odds ratios indicate the change associated with a one-point increase in these mean scores. Categorical variables are presented relative to the reference group. — indicates not applicable. AOR, adjusted odds ratio; CI, confidence interval; HL, health literacy; OR, odds ratio.

### Association between Treatment Preference and HL

Among 453 respondents with available data, 18 (4%) preferred longevity-focused care, 299 (66%) preferred comfort-focused care, and 136 (30%) were unsure (Table 1). The association between total HL and selecting unsure is presented in Table 4 (right panel). Higher total HL was associated with a lower likelihood of responding unsure (AOR, 0.63; 95% CI, 0.42 to 0.95; *P* = 0.03), as was older age (0.79; 95% CI, 0.66 to 0.95; *P* = 0.01). By contrast, HL subdomains were not significantly related to this response (functional HL: AOR, 0.83; 95% CI, 0.62 to 1.10; *P* = 0.19; communicative HL: AOR, 1.06; 95% CI, 0.68 to 1.67; *P* = 0.79; critical HL: AOR, 0.69; 95% CI, 0.44 to 1.09; *P* = 0.11; Table 4, left panel). Other covariates were not significantly associated with being unsure.

**Table 4:** Associations between health literacy and selecting unsure for treatment preferences under serious illness (*n*=453)

Variables	Multidimensional HL Model		Total HL Model	
	Unadjusted OR (95% CI), *P* Value	AOR (95% CI), *P* Value	Unadjusted OR (95% CI), *P* Value	AOR (95% CI), *P* Value
Total HL (per one-point increase)	—	—	0.65 (0.45 to 0.94), *P* = 0.02	0.63 (0.42 to 0.95), *P* = 0.03
Functional HL (per one-point increase)	0.81 (0.62 to 1.06), *P* = 0.12	0.83 (0.62 to 1.10), *P* = 0.19	—	—
Communicative HL (per one-point increase)	1.05 (0.68 to 1.64), *P* = 0.82	1.06 (0.68 to 1.67), *P* = 0.79	—	—
Critical HL (per one-point increase)	0.74 (0.48 to 1.15), *P* = 0.18	0.69 (0.44 to 1.09), *P* = 0.11	—	—
Age (per 10-yr increase)	—	0.78 (0.65 to 0.94), *P* = 0.009	—	0.79 (0.66 to 0.95), *P* = 0.01
Women (versus men)	—	0.85 (0.53 to 1.35), *P* = 0.49	—	0.84 (0.53 to 1.33), *P* = 0.45
**Comorbidities**				
Diabetes	—	1.34 (0.88 to 2.05), *P* = 0.18	—	1.34 (0.88 to 2.04), *P* = 0.18
Malignancy	—	1.34 (0.71 to 2.51), *P* = 0.37	—	1.33 (0.71 to 2.50), *P* = 0.37
**Household income**				
<3,000,000 yen (low)	—	Reference	—	Reference
3,000,000 to <5,000,000 yen (medium)	—	1.05 (0.63 to 1.75), *P* = 0.85	—	1.05 (0.63 to 1.75), *P* = 0.85
≥5,000,000 yen (high)	—	0.84 (0.47 to 1.52), *P* = 0.57	—	0.85 (0.47 to 1.53), *P* = 0.58
**Education**				
Junior high school or less	—	Reference	—	Reference
High school	—	0.75 (0.43 to 1.30), *P* = 0.31	—	0.75 (0.44 to 1.29), *P* = 0.30
University/graduate school	—	0.71 (0.32 to 1.57), *P* = 0.40	—	0.71 (0.32 to 1.56), *P* = 0.39
Others	—	0.63 (0.29 to 1.33), *P* = 0.23	—	0.63 (0.30 to 1.32), *P* = 0.22

Adjusted odds ratios were estimated using multivariable logistic regression models including all covariates simultaneously. The outcome was defined as selecting unsure versus choosing longevity-focused or comfort-focused care. Health literacy scores (range, 1–4) represent mean values; odds ratios indicate the change associated with a one-point increase in these mean scores. Categorical variables are presented relative to the reference group. — indicates not applicable. AOR, adjusted odds ratio; CI, confidence interval; HL, health literacy; OR, odds ratio.

### Reasons for Not Discussing ACP Despite Previous Consideration

Among 454 respondents, 151 (33%) reported that they had considered ACP but had not discussed it with others (QA2). The reasons for not discussing ACP are summarized in Table 5. The most frequently selected reason was lack of opportunity to discuss (71/151). A substantial proportion of participants also reported lack of knowledge (56/151) and lack of a discussion partner (18/151). By contrast, fewer participants selected reasons reflecting personal reluctance, such as not wanting to discuss it (11/151) or not feeling the need to discuss it (29/151).

**Table 5:** Reasons for not discussing advance care planning with family or their physicians (*n*=151)

Reasons	*n*
Because I do not want to discuss it	11
Because I do not feel a need to discuss it	29
Because I have not had an opportunity to discuss it	71
Because I lack sufficient knowledge to discuss it	56
Because I do not have anyone to discuss it with	18
Other reasons	7

Participants could select multiple reasons. The analysis was restricted to participants who reported not discussing advance care planning with family or their physicians.

## Discussion

In this multicenter study of patients undergoing hemodialysis, we found that higher total HL was positively associated with ACP participation. Notably, greater critical HL was specifically related to informal ACP. Higher total HL was associated with a lower likelihood of responding unsure regarding EOL care preferences. The most frequently reported reason for not participating in ACP was the lack of an opportunity to discuss it.

Our findings provide quantitative support for and extend previous qualitative studies examining the influence of HL on ACP among patients receiving dialysis. Notably, total HL—rather than individual subdomains alone—was consistently associated with informal ACP discussions, formal ACP documentation, and certainty in EOL preferences, suggesting that functional, communicative, and critical HL collectively contribute to the ACP process. The importance of functional HL is supported by Ladin *et al.* who highlighted its role in accurately understanding EOL-related terminology.^16^ For example, previous work has shown that a substantial proportion of older patients receiving dialysis misinterpret the term prognosis as diagnosis. Such misunderstandings may hinder patients from recognizing the relevance of future health trajectories, whereas adequate functional HL may enable a more accurate understanding of one's condition and facilitate engagement in ACP as a personally meaningful issue.

Communicative HL is believed to be associated with patients' ability to actively create opportunities to discuss their preferences with physicians and family members, as described by Ladin *et al.*^16^ and in other qualitative studies.^12^ For instance, some patients have been reported to actively prompt physicians—who may otherwise avoid EOL discussions—to engage in conversations about future care.^16^ This suggests that communicative HL may be related to patients' initiation of health information exchange and overcome clinician-level barriers to ACP discussions.

Critical HL has been proposed to be associated with preference-concordant care decisions by enabling patients to evaluate and integrate complex information.^16^ Our finding that treatment preferences were associated with total HL, but not with individual HL subdomains, aligns with qualitative evidence suggesting that critical appraisal of EOL information requires a foundation of both functional and communicative HL. In this sense, critical HL may be linked to patients' ability to integrate information and assess whether treatment options align with their personal values and preferences. On the one hand, passive attitudes toward medical decision making have been attributed to alignment with family or physician preferences^12,16^ or to defensive avoidance of negative emotions related to future illness and death.^12^ In this context, our finding that critical HL was associated with ACP discussion suggests that critical HL, rather than communicative HL, may be associated with more proactive engagement in patient-centered decision making for EOL care, rather than deferring decisions to family members or clinicians.

ACP can be conceptualized as a form of shared decision making, an iterative and relational process^17^ in which patients, families, and clinicians collaboratively explore and align care preferences over time.^4^ Within this framework, HL—particularly communicative and critical HL—may function not merely as a predictor of ACP participation but as a factor associated with engagement in shared decision making. From this standpoint, this study has several clinical implications for dialysis clinicians. First, providing easily understandable information about EOL care alone may be insufficient to initiate ACP discussions among patients with low functional HL, as previously suggested.^12^ Instead, our results highlight the need for approaches that enhance multidimensional HL—not only functional but also communicative and critical HL. To address low functional HL, video-based content that helps patients concretely visualize their illness trajectory and future care options may be beneficial.^18^ However, such approaches may be counterproductive for patients who have little interest in EOL care.

Second, for patients with limited communicative HL, clinicians may need to actively create opportunities for EOL discussions by carefully listening to patients' spontaneous remarks and building conversations from these cues. For example, discussion opportunities may arise when patients become aware that fellow patients receiving in-center dialysis have missed visits because of severe illness or when they report serious illness affecting someone close to them. Consistent with this, more than half of patients who had not discussed ACP cited a lack of opportunity. In addition, promoting culturally and linguistically concordant communication may be essential to support patients with limited communicative HL. Given that this study was conducted in a relatively homogenous Japanese-speaking population, further research is needed to examine the role of cultural and language concordance in more diverse settings.

Third, hospitalization because of illness may serve not only as a trigger for direct discussions with patients^4^ but also as an opportunity to involve family members in conversations about care plans in case of unexpected clinical deterioration. In Asian cultural contexts, where decision-making is often socially supported by family members,^19^ such family-inclusive discussions may help compensate for limited critical HL.^19,20^ However, previous qualitative studies have suggested that being critically ill or experiencing uremic symptoms may impair patients' cognitive and communicative capacity, making ACP discussions more difficult. Therefore, clinicians should carefully consider the timing of such discussions and, when possible, initiate them when patients are clinically stable and able to meaningfully engage.

Finally, addressing HL requires a multilevel and multidimensional approach that considers not only individual capabilities but also broader social determinants of health.^21^ Factors such as transportation, insurance coverage, financial constraints, and caregiving responsibilities may limit patients' ability to engage in ACP discussions and to act on their preferences. These structural barriers are closely linked to treatment adherence and overall well-being^22,23^ and should be considered integral to patient-centered ACP implementation. Accordingly, interventions to promote ACP should extend beyond individual-level education and incorporate system-level strategies that facilitate equitable patient participation.

Beyond individual-level approaches, our findings suggest the importance of organizational and team-based strategies to facilitate ACP discussions. Multidisciplinary teams—including physicians, nurses, and social workers—could incorporate structured prompts for ACP at key clinical events, such as dialysis initiation, hospitalization, or changes in clinical status. Routine screening for HL or communication needs, even using brief pragmatic tools, may help tailor these discussions to individual patients in routine clinical workflows. In this context, organizational HL^24^—defined as the extent to which health care systems enable patients to access, understand, and use health information—may be important for translating individual capacities into meaningful ACP engagement.

This study has several limitations. First, owing to its cross-sectional design, causal relationships between HL and ACP participation cannot be established. This study was conducted in Japan, where formal ACP practices remain relatively uncommon and are structurally and culturally constrained, potentially influencing both the overall prevalence of ACP participation and the observed associations, particularly for formal ACP. Second, we did not assess participants' knowledge or understanding of ACP; therefore, we could not determine whether HL was associated with ACP participation through greater ACP-related knowledge or comprehension. Third, a small proportion of participants (*n*=15) were receiving combination therapy with hemodialysis and peritoneal dialysis, precluding stratified analyses by dialysis modality. Previous studies have suggested that HL may differ between in-center and home-based dialysis populations.^25^ Similarly, the number of participants with formal ACP participation was small (*n*=24), resulting in wide CIs and potential instability of the regression estimates. This low prevalence also limited our statistical power to detect associations with individual HL subdomains. Therefore, these findings should be interpreted with caution, and larger studies are warranted to confirm these associations. Fourth, unmeasured factors—such as social support, family structure (including marital status), the duration and quality of patient–provider relationships (including health care professional support), hospitalization history, and depressive symptoms—may have affected the observed associations between HL and ACP participation. Previous studies have reported that depressive symptoms are positively associated with ACP participation^26^ and inversely associated with HL^27^; therefore, failure to adjust for depressive symptoms may have led to an underestimation of the association between HL and ACP participation. We also did not assess adherence to prescribed hemodialysis treatment (*e.g*., missed or shortened sessions), which has been associated with HL in previous studies.^28^ Although adherence may be related to engagement in ACP, its role in the causal pathway between HL and ACP remains uncertain. In Japan, however, nonadherence to hemodialysis treatment is relatively uncommon—for example, the prevalence of skipping at least one dialysis session per month has been reported to be approximately 0.6%^29^—suggesting that its impact on our findings is likely limited. Nevertheless, future studies incorporating adherence measures would provide a more comprehensive understanding. Fifth, questionnaires were completed at home without standardized support for participants with limited functional HL. Although participants were instructed to respond independently, some may have received assistance (*e.g*., reading support), which could have influenced responses in unknown directions. In addition, individuals with very low functional HL may have been less likely to participate in the study, potentially leading to selection bias and an underestimation of the association between HL and ACP engagement. However, given the high literacy rate in Japan, the proportion of participants unable to comprehend the questionnaire is likely to have been small. Finally, HL was assessed using self-reported measures, which may be subject to social desirability bias and may reflect perceived rather than actual capacity, potentially introducing measurement error.

In conclusion, this study demonstrated that multidimensional HL is associated with a greater likelihood of ACP participation and reduced uncertainty regarding EOL care preferences among patients undergoing dialysis. Critical HL was particularly associated with informal ACP. A lack of opportunities for discussion was the most reported barrier to ACP, suggesting the importance of clinician support. Clinician support that extends beyond functional HL, together with family involvement, may contribute to the initiation and progression of ACP participation by supporting communicative and critical HL.

## Supplementary Material

## Data Availability

Original data generated for the study will be made available upon reasonable request to the corresponding author. Data Type: Observational Data. Reason for Restricted Access: Access to the dataset is restricted in accordance with national ethical guidelines for medical and health research involving human subjects. The informed consent obtained from participants was limited to use within the approved study objectives and did not include consent for unrestricted public sharing of individual-level data. To protect participant privacy and comply with institutional ethics approval, access to the dataset is therefore restricted.

## Declarative Statements

This study includes clinical experimentation and received Institutional Review Board or Ethics Committee approval. All patients provided written informed consent. This study includes clinical experimentation and complies with the Declaration of Helsinki.

## Supplemental Material

Supplemental Item 1. The questionnaire on ACP-related measures.

Supplemental Table 1. The functional communicative critical HL scale.

Supplemental Item 2. A brief description of covariate definitions.
