# Supplementary material for: Association of Formal and Informal Advance Care Planning with Multidimensional Health Literacy in Patients Undergoing Hemodialysis
Source: Kidney360. 2026 May 26;7(8):1825–34. doi: 10.34067/KID.0000001243 (PMC13567901; doi:10.34067/KID.0000001243)
Supplement: Supplementary file 1 [file kidney360-7-1825-s001.pdf]

ASN American Society of Nephrology
JASN Journal of the American Society of Nephrology
KIDNEY360 Accessing Our World From Every Angle
CJASN Clinical Journal of the American Society of Nephrology

ASN Journal Disclosure Form

As per ASN journal policy, I have disclosed any financial relationships or commitments I have held in the past 36 months as included below. I have listed my Current Employer below to indicate there is a relationship requiring disclosure. If no relationship exists, my Current Employer is not listed.

T. Aita has nothing to disclose.

I understand that the information above will be published within the journal article, if accepted, and that failure to comply and/or to accurately and completely report the potential financial conflicts of interest could lead to the following: 1) Prior to publication, article rejection, or 2) Post-publication, sanctions ranging from, but not limited to, issuing a correction, reporting the inaccurate information to the authors' institution, banning authors from submitting work to ASN journals for varying lengths of time, and/or retraction of the published work.

Name: Tetsuro Aita
Manuscript ID: K360-2026-000135R1
Manuscript Title: Association of Formal and Informal Advance Care Planning with Multidimensional Health Literacy in Patients Undergoing Hemodialysis
Date of Completion: April 14, 2026
Disclosure Updated Date: April 14, 2026

ASN
American Society of Nephrology
JASN
Journal of the American Society of Nephrology
KIDNEY360
Accessing Our World From Every Angle
CJASN
Clinical Journal of the American Society of Nephrology

ASN Journal Disclosure Form

As per ASN journal policy, I have disclosed any financial relationships or commitments I have held in the past 36 months as included below. I have listed my Current Employer below to indicate there is a relationship requiring disclosure. If no relationship exists, my Current Employer is not listed.

R. Inanaga reports the following:
Honoraria: Vantive Japan

I understand that the information above will be published within the journal article, if accepted, and that failure to comply and/or to accurately and completely report the potential financial conflicts of interest could lead to the following: 1) Prior to publication, article rejection, or 2) Post-publication, sanctions ranging from, but not limited to, issuing a correction, reporting the inaccurate information to the authors' institution, banning authors from submitting work to ASN journals for varying lengths of time, and/or retraction of the published work.

Name: Ryohei Inanaga
Manuscript ID: K360-2026-000135R1
Manuscript Title: Association of Formal and Informal Advance Care Planning with Multidimensional Health Literacy in Patients Undergoing Hemodialysis
Date of Completion: April 14, 2026
Disclosure Updated Date: April 14, 2026

ASN
American Society of Nephrology
JASN
Journal of the American Society of Nephrology
KIDNEY360
Accessing Our World From Every Angle
CJASN
Clinical Journal of the American Society of Nephrology

ASN Journal Disclosure Form

As per ASN journal policy, I have disclosed any financial relationships or commitments I have held in the past 36 months as included below. I have listed my Current Employer below to indicate there is a relationship requiring disclosure. If no relationship exists, my Current Employer is not listed.

Y. Kanakubo reports the following:
Employer: The Jikei University School of Medicine, Kameda Family Clinic Tateyama; Research Funding: MSD; and Other Interests or Relationships: YK is a member of the committee on diversity promotion in Japan Primary Care Association (JPCA), the committee on sexual health in JPCA, and the Gender Equality Promotion Committee in Chiba Medical Association. YK serves as a member of the board of Nijiiro Doctors, an organization to promote awareness and education within the medical community regarding LGBTQ issues. As a member of Nijiro Doctors, YK works with Novartis Pharma K.K. on projects but does not receive personal board compensation.

I understand that the information above will be published within the journal article, if accepted, and that failure to comply and/or to accurately and completely report the potential financial conflicts of interest could lead to the following: 1) Prior to publication, article rejection, or 2) Post-publication, sanctions ranging from, but not limited to, issuing a correction, reporting the inaccurate information to the authors' institution, banning authors from submitting work to ASN journals for varying lengths of time, and/or retraction of the published work.

Name: Yusuke Kanakubo
Manuscript ID: K360-2026-000135R1
Manuscript Title: Association of Formal and Informal Advance Care Planning with Multidimensional Health Literacy in Patients Undergoing Hemodialysis
Date of Completion: April 14, 2026
Disclosure Updated Date: April 14, 2026

ASN American Society of Nephrology
JASN Journal of the American Society of Nephrology
KIDNEY360 Accessing Our World From Every Angle
CJASN Clinical Journal of the American Society of Nephrology

ASN Journal Disclosure Form

As per ASN journal policy, I have disclosed any financial relationships or commitments I have held in the past 36 months as included below. I have listed my Current Employer below to indicate there is a relationship requiring disclosure. If no relationship exists, my Current Employer is not listed.

A. Kawaji reports the following:
Employer: Kameda General Hospital

I understand that the information above will be published within the journal article, if accepted, and that failure to comply and/or to accurately and completely report the potential financial conflicts of interest could lead to the following: 1) Prior to publication, article rejection, or 2) Post-publication, sanctions ranging from, but not limited to, issuing a correction, reporting the inaccurate information to the authors' institution, banning authors from submitting work to ASN journals for varying lengths of time, and/or retraction of the published work.

Name: Atsuro Kawaji
Manuscript ID: K360-2026-000135R1
Manuscript Title: Association of Formal and Informal Advance Care Planning with Multidimensional Health Literacy in Patients Undergoing Hemodialysis
Date of Completion: April 14, 2026
Disclosure Updated Date: April 14, 2026

ASN
American Society of Nephrology
JASN
Journal of the American Society of Nephrology
KIDNEY360
Accessing Our World From Every Angle
CJASN
Clinical Journal of the American Society of Nephrology

ASN Journal Disclosure Form

As per ASN journal policy, I have disclosed any financial relationships or commitments I have held in the past 36 months as included below. I have listed my Current Employer below to indicate there is a relationship requiring disclosure. If no relationship exists, my Current Employer is not listed.

N. Kurita reports the following:
Employer: Department of Preventive Medicine, Tokai University School of Medicine; Consultancy: GlaxoSmithKline K.K.; Kyowa Kirin Co., Ltd.; Honoraria: Eisai Co., Ltd.; Taisho Pharmaceutical Co., Ltd.; Kyowa Kirin Co., Ltd.; GlaxoSmithKline K.K.; Takeda Pharmaceutical Co., Ltd.; Kissei Pharmaceutical Co., Ltd.; Vantive Japan; and Speakers Bureau: Eisai Co., Ltd.; Taisho Pharmaceutical Co., Ltd.; Kyowa Kirin Co., Ltd.; GlaxoSmithKline K.K.; Takeda Pharmaceutical Co., Ltd.; Kissei Pharmaceutical Co., Ltd.; Vantive Japan.

I understand that the information above will be published within the journal article, if accepted, and that failure to comply and/or to accurately and completely report the potential financial conflicts of interest could lead to the following: 1) Prior to publication, article rejection, or 2) Post-publication, sanctions ranging from, but not limited to, issuing a correction, reporting the inaccurate information to the authors' institution, banning authors from submitting work to ASN journals for varying lengths of time, and/or retraction of the published work.

Name: Noriaki Kurita
Manuscript ID: K360-2026-000135R3
Manuscript Title: Association of Formal and Informal Advance Care Planning with Multidimensional Health Literacy in Patients Undergoing Hemodialysis
Date of Completion: May 12, 2026
Disclosure Updated Date: April 14, 2026

ASN American Society of Nephrology · JASN Journal of the American Society of Nephrology · KIDNEY360 Accessing Our World From Every Angle · CJASN Clinical Journal of the American Society of Nephrology

ASN Journal Disclosure Form

As per ASN journal policy, I have disclosed any financial relationships or commitments I have held in the past 36 months as included below. I have listed my Current Employer below to indicate there is a relationship requiring disclosure. If no relationship exists, my Current Employer is not listed.

M. Matsunami reports the following:
Employer: Kameda Medical Center; Honoraria: Masatoshi Matsunami received payments for speaking and educational events from Astellas Pharma Inc. and Baxter Co., Ltd.; and Speakers Bureau: Masatoshi Matsunami received payments for speaking and educational events from Astellas Pharma Inc. and Baxter Co., Ltd.

I understand that the information above will be published within the journal article, if accepted, and that failure to comply and/or to accurately and completely report the potential financial conflicts of interest could lead to the following: 1) Prior to publication, article rejection, or 2) Post-publication, sanctions ranging from, but not limited to, issuing a correction, reporting the inaccurate information to the authors' institution, banning authors from submitting work to ASN journals for varying lengths of time, and/or retraction of the published work.

Name: Masatoshi Matsunami
Manuscript ID: K360-2026-000135R1
Manuscript Title: Association of Formal and Informal Advance Care Planning with Multidimensional Health Literacy in Patients Undergoing Hemodialysis
Date of Completion: April 14, 2026
Disclosure Updated Date: April 14, 2026

ASN
American Society of Nephrology
JASN
Journal of the American Society of Nephrology
KIDNEY360
Accessing Our World From Every Angle
CJASN
Clinical Journal of the American Society of Nephrology

ASN Journal Disclosure Form

As per ASN journal policy, I have disclosed any financial relationships or commitments I have held in the past 36 months as included below. I have listed my Current Employer below to indicate there is a relationship requiring disclosure. If no relationship exists, my Current Employer is not listed.

T. Okada has nothing to disclose.

I understand that the information above will be published within the journal article, if accepted, and that failure to comply and/or to accurately and completely report the potential financial conflicts of interest could lead to the following: 1) Prior to publication, article rejection, or 2) Post-publication, sanctions ranging from, but not limited to, issuing a correction, reporting the inaccurate information to the authors' institution, banning authors from submitting work to ASN journals for varying lengths of time, and/or retraction of the published work.

Name: Tadao Okada
Manuscript ID: K360-2026-000135R3
Manuscript Title: Association of Formal and Informal Advance Care Planning with Multidimensional Health Literacy in Patients Undergoing Hemodialysis
Date of Completion: May 12, 2026
Disclosure Updated Date: April 14, 2026

ASN Journal Disclosure Form

As per ASN journal policy, I have disclosed any financial relationships or commitments I have held in the past 36 months as included below. I have listed my Current Employer below to indicate there is a relationship requiring disclosure. If no relationship exists, my Current Employer is not listed.

T. Suzuki reports the following:
Employer: Department of Nephrology, Kameda Medical Center

I understand that the information above will be published within the journal article, if accepted, and that failure to comply and/or to accurately and completely report the potential financial conflicts of interest could lead to the following: 1) Prior to publication, article rejection, or 2) Post-publication, sanctions ranging from, but not limited to, issuing a correction, reporting the inaccurate information to the authors' institution, banning authors from submitting work to ASN journals for varying lengths of time, and/or retraction of the published work.

Name: Tomo Suzuki
Manuscript ID: K360-2026-000135R1
Manuscript Title: Association of Formal and Informal Advance Care Planning with Multidimensional Health Literacy in Patients Undergoing Hemodialysis
Date of Completion: April 14, 2026
Disclosure Updated Date: April 14, 2026

ASN American Society of Nephrology | JASN Journal of the American Society of Nephrology | KIDNEY360 Accessing Our World From Every Angle | CJASN Clinical Journal of the American Society of Nephrology

ASN Journal Disclosure Form

As per ASN journal policy, I have disclosed any financial relationships or commitments I have held in the past 36 months as included below. I have listed my Current Employer below to indicate there is a relationship requiring disclosure. If no relationship exists, my Current Employer is not listed.

T. Toida reports the following:
Employer: Toho University Ohashi Medical Center; and Speakers Bureau: Torii Pharmaceutical Co., Ltd., Ono Pharmaceutical Co., Ltd., Kyowa Kirin Co., Ltd., AstraZeneca K.K., Nobelpharma Co., Ltd., and Nippon Boehringer Ingelheim Co., Ltd.

I understand that the information above will be published within the journal article, if accepted, and that failure to comply and/or to accurately and completely report the potential financial conflicts of interest could lead to the following: 1) Prior to publication, article rejection, or 2) Post-publication, sanctions ranging from, but not limited to, issuing a correction, reporting the inaccurate information to the authors' institution, banning authors from submitting work to ASN journals for varying lengths of time, and/or retraction of the published work.

Name: Tatsunori Toida
Manuscript ID: K360-2026-000135R1
Manuscript Title: Association of Formal and Informal Advance Care Planning with Multidimensional Health Literacy in Patients Undergoing Hemodialysis
Date of Completion: April 14, 2026
Disclosure Updated Date: April 14, 2026

ASN American Society of Nephrology JASN Journal of the American Society of Nephrology KIDNEY360 Accessing Our World From Every Angle CJASN Clinical Journal of the American Society of Nephrology

ASN Journal Disclosure Form

As per ASN journal policy, I have disclosed any financial relationships or commitments I have held in the past 36 months as included below. I have listed my Current Employer below to indicate there is a relationship requiring disclosure. If no relationship exists, my Current Employer is not listed.

T. Toishi reports the following:
Employer: Kameda medical center

I understand that the information above will be published within the journal article, if accepted, and that failure to comply and/or to accurately and completely report the potential financial conflicts of interest could lead to the following: 1) Prior to publication, article rejection, or 2) Post-publication, sanctions ranging from, but not limited to, issuing a correction, reporting the inaccurate information to the authors' institution, banning authors from submitting work to ASN journals for varying lengths of time, and/or retraction of the published work.

Name: Takumi Toishi
Manuscript ID: K360-2026-000135R3
Manuscript Title: Association of Formal and Informal Advance Care Planning with Multidimensional Health Literacy in Patients Under
Date of Completion: May 12, 2026
Disclosure Updated Date: May 12, 2026

ASN American Society of Nephrology | JASN Journal of the American Society of Nephrology | KIDNEY360 Accessing Our World From Every Angle | CJASN Clinical Journal of the American Society of Nephrology

ASN Journal Disclosure Form

As per ASN journal policy, I have disclosed any financial relationships or commitments I have held in the past 36 months as included below. I have listed my Current Employer below to indicate there is a relationship requiring disclosure. If no relationship exists, my Current Employer is not listed.

M. Ukai reports the following:
Employer: Kameda Family Clinic Tateyama

I understand that the information above will be published within the journal article, if accepted, and that failure to comply and/or to accurately and completely report the potential financial conflicts of interest could lead to the following: 1) Prior to publication, article rejection, or 2) Post-publication, sanctions ranging from, but not limited to, issuing a correction, reporting the inaccurate information to the authors' institution, banning authors from submitting work to ASN journals for varying lengths of time, and/or retraction of the published work.

Name: Mamiko Ukai
Manuscript ID: K360-2026-000135R1
Manuscript Title: Association of Formal and Informal Advance Care Planning with Multidimensional Health Literacy in Patients Undergoing Hemodialysis
Date of Completion: April 14, 2026
Disclosure Updated Date: April 14, 2026

ASN Journal Disclosure Form

As per ASN journal policy, I have disclosed any financial relationships or commitments I have held in the past 36 months as included below. I have listed my Current Employer below to indicate there is a relationship requiring disclosure. If no relationship exists, my Current Employer is not listed.

M. Yu reports the following:
Employer: Munakata clinic

I understand that the information above will be published within the journal article, if accepted, and that failure to comply and/or to accurately and completely report the potential financial conflicts of interest could lead to the following: 1) Prior to publication, article rejection, or 2) Post-publication, sanctions ranging from, but not limited to, issuing a correction, reporting the inaccurate information to the authors' institution, banning authors from submitting work to ASN journals for varying lengths of time, and/or retraction of the published work.

Name: Munakata Yu
Manuscript ID: K360-2026-000135R1
Manuscript Title: Association of Formal and Informal Advance Care Planning with Multidimensional Health Literacy in Patients Undergoing Hemodialysis
Date of Completion: April 15, 2026
Disclosure Updated Date: April 15, 2026
