# Supplementary material for: Association of Formal and Informal Advance Care Planning with Multidimensional Health Literacy in Patients Undergoing Hemodialysis
Source: Kidney360. 2026 May 26;7(8):1825–34. doi: 10.34067/KID.0000001243 (PMC13567901; doi:10.34067/KID.0000001243)
Supplement: Supplementary file 2 [file kidney360-7-1825-s002.pdf]

Supplemental Information

Supplemental Item 1: The questionnaire on Advance Care Planning related measures

Instruction

"This section asks about your thoughts regarding your health care if you were to become very sick in the future."

Assessment of ACP Participation (Question A)

"Have you thought about the kinds of treatments that you would want or not want if you were to become very sick and were unable to speak for yourself? (Check **all that apply**)"

Response options:

QA1: I have not thought about this

QA2: I have thought about this but have not talked to anyone about it

QA3: I have talked about this with a friend or family member but have not signed official papers

QA4: I have talked about this with a doctor or other healthcare provider but have not signed official papers

QA5: I have signed official papers

Responses to Question A were categorized for analysis as follows:

No ACP: QA1 or QA2

Informal ACP: QA3 or QA4 (discussion without formal documentation)

Formal ACP: QA5 (completion of advance directives)

Treatment Preference (Question B)

"If you were to become very sick in the future and were unable to speak for yourself, would you prefer a plan of medical care that focuses on extending life as much as possible, even if it means

having more pain and discomfort, or would you prefer medical care that focuses on relieving pain and discomfort as much as possible, even if that means not living as long?"

Participants were instructed to select **one** response.

Response options:

QB1: Extending life, even if that means having more pain and discomfort

QB2: Relieving pain and discomfort as much as possible, even if that means not living as long

QB3: I am not sure which I would choose

Responses to Question B were categorized for analysis as follows:

Longevity-focused care: QB1 ("Extending life, even if that means having more pain and discomfort")

Comfort-focused care: QB2 ("Relieving pain and discomfort as much as possible, even if that means not living as long")

Unsure: QB3 ("I am not sure which I would choose")

Reasons for Not Discussing ACP (Question C)

Instruction (branching condition)

Participants who responded "I have thought about this but have not talked to anyone about it" (QA2) in Question A were asked the following:

"What are the reasons you have not discussed your preferences for future medical care? (Select all that apply.)"

Response options:

QC1: Because I do not want to discuss it

QC2: Because I do not feel the need to discuss it

QC3: Because I have not had an opportunity to discuss it

QC4: Because I do not know what to discuss due to lack of knowledge

QC5: Because I do not have anyone to discuss it with

QC6: Other reasons

Supplemental Table 1. The Functional Communicative Critical Health Literacy Scale

	Question
Instruction sentence	あなたは、この 1 年間に、病院や薬局からもらう説明書やパンフレットを読む際、次のようなことがありましたか。最も当てはまる番号に○をしてください。<br>English: "In reading instructions or leaflets from hospitals/pharmacies, you. . .".
Question 1	字が細かくて、読みにくい（メガネなどをかけた状態でも）<br>(English: "found that the print was too small to read.")
Question 2	読めない漢字や知らない言葉がある。<br>(English: "found characters and words that you did not know")
Question 3	内容が難しくて分かりにくい<br>(English: "found that the content was too difficult.")
Question 4	読むのに時間がかかる<br>(English: "needed a long time to read and understand them.")
Question 5	誰かに代わりに読んで教えてもらう<br>(English: "needed someone to help you read them.")
Instruction sentence	腎臓病と診断されてから、腎臓病やその治療・健康法に関することについて、以下のようなことをしましたか。最も当てはまる番号に○をしてください。<br>(English: "Since having kidney disease you have. . .")
Question 1	いろいろなところから知識や情報を集めた。<br>(English: "collected information from various sources.")
Question 2	たくさんある知識や情報から、自分の求めるものを選び出した。<br>(English: "extracted the information you wanted.")
Question 3	自分が見聞きした知識や情報を、理解できた。<br>(English: "understood the obtained information.")
Question 4	病気についての自分の意見や考えを、医師や身近な人に伝えた。<br>(English: "communicated your thoughts about your illness to someone.")
Question 5	見聞きした知識や情報をもとに、実際に生活を変えてみた。<br>(English: "applied the obtained information to your daily life..")
Question 6	見聞きした知識や情報が、自分にもあてはまるかどうか考えた。

	(English: "considered whether the information was applicable to your situation.")
Question 7	見聞きした知識や情報の信頼性に疑問をもった。<br>(English: "considered the credibility of the information.")
Question 8	見聞きした知識や情報が正しいかどうか聞いたり、調べたりした。<br>(English: "checked whether the information was valid and reliable.")
Question 9	病気や治療法などを自分で決めるために調べた。<br>(English: "collected information to make health-related decisions.")
Response options for questions	全くなかった/あまりなかった/時々あった/よくあった<br>(English: "never / rarely / sometimes / often")

The English-translated version [1] is also provided for each item and response.

Reference

1. Ishikawa H, Takeuchi T, Yano E. Measuring functional, communicative, and critical health literacy among diabetic patients. *Diabetes Care*. 2008;31(5):874–879. doi: 10.2337/dc07-1932.

Supplemental Item 2: A brief description of covariate definitions

Comorbidities were assessed by the treating dialysis physicians using a predefined case report form, based on components of the Charlson Comorbidity Index.

Diabetes mellitus was categorized as follows:

0 = no diabetes;

1 = diabetes managed with diet alone;

2 = diabetes requiring pharmacological treatment without end-organ complications;

3 = diabetes with complications (retinopathy, neuropathy, or nephropathy).

For analysis, diabetes was defined as present if the score was ≥1.

Malignancy was defined as present if any of the following conditions were met:

(1) solid tumor (score ≥1), categorized as no tumor or disease-free for ≥5 years (0), non-metastatic tumor (1), or metastatic tumor (2);

(2) leukemia (acute or chronic; 0 = no, 1 = yes); or

(3) lymphoma, including Hodgkin lymphoma, Waldenström macroglobulinemia, and multiple myeloma (0 = no, 1 = yes).

Household income and educational attainment were self-reported questionnaires.

Participants selected their total annual household income (before tax, including pensions) from predefined categories:

<1,000,000 yen; 1,000,000–<3,000,000 yen; 3,000,000–<5,000,000 yen; 5,000,000–<8,000,000 yen; 8,000,000–<10,000,000 yen; 10,000,000–<15,000,000 yen; or ≥15,000,000 yen.

For analysis, these categories were grouped into three levels: low (<3,000,000 yen), medium (3,000,000–<5,000,000 yen), and high (≥5,000,000 yen).

Educational attainment was categorized as:

(1) junior high school or less;

(2) high school;

(3) vocational school or junior college;

(4) university or graduate school;

(5) other.

Vocational school/junior college and “other” were combined into a single “Others” category for analysis.
